# A green-light inducible lytic system for cyanobacterial cells

**DOI:** 10.1186/1754-6834-7-56

**Published:** 2014-04-09

**Authors:** Kotone Miyake, Koichi Abe, Stefano Ferri, Mitsuharu Nakajima, Mayumi Nakamura, Wataru Yoshida, Katsuhiro Kojima, Kazunori Ikebukuro, Koji Sode

**Affiliations:** 1 Department of Biotechnology, Graduate School of Engineering, Tokyo University of Agriculture & Technology, 2-24-16 Naka-cho, Koganei, Tokyo 184-8588, Japan; 2 JST, CREST, 2-24-16 Naka-cho, Koganei, Tokyo 184-8588, Japan

**Keywords:** Cyanobacteria, Self-lysis, Two-component system, T4 bacteriophage, Synthetic biology

## Abstract

**Background:**

Cyanobacteria are an attractive candidate for the production of biofuel because of their ability to capture carbon dioxide by photosynthesis and grow on non-arable land. However, because huge quantities of water are required for cultivation, strict water management is one of the greatest issues in algae- and cyanobacteria-based biofuel production. In this study, we aim to construct a lytic cyanobacterium that can be regulated by a physical signal (green-light illumination) for future use in the recovery of biofuel related compounds.

**Results:**

We introduced T4 bacteriophage-derived lysis genes encoding holin and endolysin under the control of the green-light regulated *cpcG2* promoter in *Synechocystis* sp. PCC 6803. When cells harboring the lysis genes were illuminated with both red and green light, we observed a considerable decrease in growth rate, a significant increase in cellular phycocyanin released in the medium, and a considerable fraction of dead cells. These effects were not observed when these cells were illuminated with only red light, or when cells not containing the lysis genes were grown under either red light or red and green light.

These results indicate that our constructed green-light inducible lytic system was clearly induced by green-light illumination, resulting in lytic cells that released intracellular phycocyanin into the culture supernatant. This property suggests a future possibility to construct photosynthetic genetically modified organisms that are unable to survive under sunlight exposure. Expression of the self-lysis system with green-light illumination was also found to greatly increase the fragility of the cell membrane, as determined by subjecting the induced cells to detergent, osmotic-shock, and freeze-thaw treatments.

**Conclusions:**

A green-light inducible lytic system was constructed in *Synechocystis* sp. PCC 6803. The engineered lytic cyanobacterial cells should be beneficial for the recovery of biofuels and related compounds from cells with minimal effort and energy, due to the fragile nature of the induced cells. Furthermore, the use of light-sensing two-component systems to regulate the expression of exogenous genes in cyanobacteria promises to replace conventional chemical inducers in many bioprocess applications, impacting the limiting water management issues.

## Background

There is an increasingly urgent search for sustainable and renewable energy alternatives to fossil fuels [1]. Cyanobacteria are an attractive candidate for the production of biofuel because of their ability to capture carbon dioxide by photosynthesis and grow on non-arable land, which does not compete with terrestrial agricultural crops for land or water [2]. Furthermore, many different synthetic biology tools are now available for the convenient genetic engineering of cyanobacteria [3-7]. Although many strategies are now being reported for producing valuable compounds in genetically engineered cyanobacteria, an efficient method of collecting the synthesized compounds remains to be developed. Among the various biofuel-related products expected to be produced in cyanobacteria, there is a particular focus on the accumulation of intracellular lipids [8]. A successful downstream process to recover intracellular lipids will require disrupting the native structure of the cyanobacterial cell prior to extraction with solvents. Mechanical methods have generally been used to disrupt cells, such as by cell homogenizer, bead mill, ultrasound, French press, and autoclave. Non-mechanical methods, such as freeze-thaw, organic solvents, detergents, osmotic shock, acid, base, and enzyme reactions have also been employed. The use of supercritical methanol to convert wet algae to biodiesel has also been recently reported [9]. However, energy consumption is a concern for mechanical methods, while non-mechanical methods require additional steps for removing the chemicals, resulting in extra costs. More efficient and cost-effective methods must therefore be designed.

An additional advantage of using algae for biofuel production is the possibility of using water that is unsuitable for land crops, such as saline water from aquifers and seawater, especially when using marine-derived strains. At the same time, strict water management is one of the greatest issues in biofuel production. Considering the use of huge quantities of water required for algal cultivation, water recycling is an essential feature. Most of the genetic tools developed for cyanobacterial bioprocesses are based on chemical induction. Since the removal of such chemicals is required for recycling the water for cultivation, chemical induction methods are not appropriate for industrial processes.

Liu and Curtiss reported pioneer work on a programmed cell lysis system for the cyanobacterium *Synechocystis* sp. PCC 6803 (hereafter *Synechocystis*) [10]. This system consists of a constitutively expressed P22 bacteriophage-derived endolysin and auxiliary enzyme, and P22 holin expressed under a nickel-ion inducible promoter, which originates from the *Synechocystis* nrsBACD operon involved in Ni^2+^ resistance [11]. This lysis system was somewhat successful in that the expression of lysis genes could be regulated inside cyanobacterial cells, and therefore contributes to reducing the energy required for extraction of the intracellular product. However, the use of a nickel-ion induction system limits its feasible application for future biofuel production processes [12].

**Figure 1:**
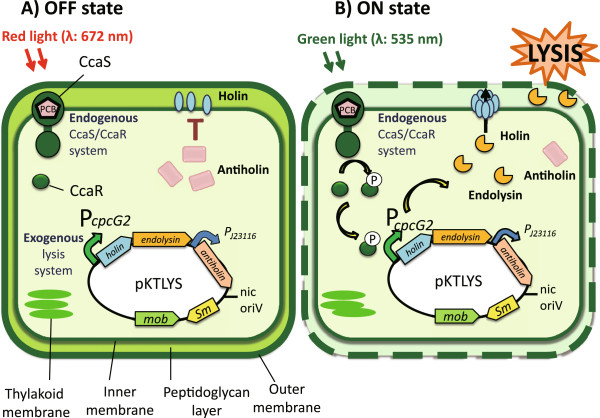
**Regulation of cyanobacterial lytic system by green light.** Schematic diagram of a novel system to prepare lytic cyanobacterial cells by physical signal, green-light illumination. The lysis system is composed of the CcaS/CcaR green-light regulated two-component system derived from *Synechocystis*, combined with T4 phage-derived lysis genes under the CcaS/CcaR-regulated *cpcG2* promoter. CcaS is a sensor histidine kinase that activates its cognate response regulator, CcaR, under green light to induce the expression of the *cpcG2* gene. Under red light, CcaS is in an ‘off-state’ and expression of the target gene is not activated **(A)**. However under green light, CcaS is in an ‘on-state’, resulting in activation of CcaR by phosphorylation and a concomitant induction of the target gene expression **(B)**. Holin forms a ‘tunnel’ in the plasma membrane, which provides endolysin access to the peptidoglycan and allows it to enzymatically break it down, leading to lysis. Antiholin, which is regulated by a weak constitutive promoter, blocks holin and prevents premature lysis due to low background levels of endolysin.

We propose a novel process to prepare lytic cyanobacteria that can be regulated by a physical signal, green-light illumination, for future use in the recovery of biofuel-related compounds (Figure 1). *Synechocystis* possesses light responsive two-component systems, such as the CcaS/CcaR two-component system, which responds to red/green light [13]. The cyanobacteriochrome CcaS and response regulator CcaR regulate the expression of *cpcG2*, encoding a phycobilisome linker protein. The expression of *cpcG2* is up-regulated by green-orange light (550-600 nm) and down-regulated by red light (672 nm). By placing T4 phage-derived lysis genes under the control of the *cpcG2* promoter on a broad host range vector and introducing it into *Synechocystis*, we designed a green-light inducible lytic cyanobacteria for future use in the recovery of biofuel-related compounds.

## Results and discussion

### Green-light induction of cell lysis

The T4 phage-derived lysis genes encoding holin, endolysin, and antiholin [14-17] were introduced into *Synechocystis* on the broad host range vector pKT230 [18], which has been utilized for the recombinant expression in both freshwater and marine cyanobacterial strains [19-24]. To link the lysis genes to the endogenous CcaS/CcaR two-component system for green-light regulation, the region upstream of the *Synechocystis cpcG2* gene was amplified by PCR and inserted upstream of the T4 holin and endolysin genes (Figure 1). Antiholin was inserted downstream of a weak constitutive promoter to prevent premature lysis from leaky expression of the holin and endolysin genes prior to induction (data not shown). *Synechocystis* transformed with the pKT230 empty vector (*Synechocystis*/pKT230) or pKT230 harboring the above lysis cassette (*Synechocystis*/pKTLYS) were grown at 30°C under red-light illumination (660 nm, 20 μmol m^-2^ s^-1^) alone or under simultaneous red- and green-light illumination (520 nm, 20 μmol m^-2^ s^-1^).

**Figure 2:**
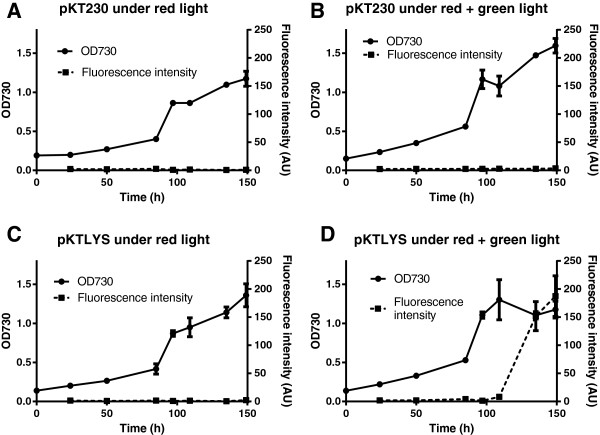
**Growth curves and supernatant fluorescence of*Synechocystis*cultures.** Growth curves (circles with solid line) and supernatant fluorescence intensity (squares with dashed line; excitation: 620 nm, emission: 650 nm), representing leaked phycocyanin, of *Synechocystis*/pKT230 **(A and B)** and *Synechocystis*/pKTLYS **(C and D)**. The cells were grown under either continuous red-light illumination **(A and C)** or under red light for 84 hours followed by simultaneous red- and green-light illumination **(B and D)**.

Figure 2 shows the cell growth of *Synechocystis*/pKT230 (Figure 2A, B) and *Synechocystis*/pKTLYS (Figure 2C, D) under continuous red-light illumination (Figure 2A, C) or under red-light illumination for 84 hours followed by simultaneous red- and green-light illumination (Figure 2B, D). In order to investigate cell lysis, phycocyanin-derived fluorescence of the culture supernatant was also monitored as an indicator of the leakage of intracellular components [25]. Cell growth reached the early logarithmic phase after approximately 80 hours of cultivation under red-light illumination, with no significant difference in growth rate between *Synechocystis*/pKT230 and *Synechocystis*/pKTLYS. In both cultures, no phycocyanin-derived fluorescence was observed under red-light illumination. After 84 hours of incubation under red-light illumination, some cells were transferred for a further 64 hours under simultaneous red- and green-light illumination (Figure 2B, D). The additional green-light illumination did not cause any significant change in the cellular growth of *Synechocystis*/pKT230 (Figure 2B) when compared with cultivation under red-light illumination alone (Figure 2A). In contrast, the culture of *Synechocystis*/pKTLYS under simultaneous red- and green-light illumination showed a considerable decrease in growth rate at about 48 hours after starting green-light illumination (Figure 2D). The cell growth of *Synechocystis*/pKTLYS under only red-light illumination (Figure 2C) was similar with that of the *Synechocystis*/pKT230 cultures (Figure 2A, B). More remarkably, the drastic increase in the phycocyanin fluorescence in the supernatant was observed only in *Synechocystis*/pKTLYS under simultaneous red- and green-light illumination, at about 64 hours after starting green-light illumination. Considering that no phycocyanin-derived fluorescence was observed from the culture supernatant of *Synechocystis*/pKT230 under both illumination conditions (Figure 2A, B), as well as from the culture supernatant of *Synechocystis*/pKTLYS under only red-light illumination (Figure 2C), the observed fluorescence increase from green-light illuminated *Synechocystis*/pKTLYS was due to the induction of lysis genes by the introduced functional green-light regulated promoter region, causing the partial leakage of intracellular proteins including phycocyanin. The observed decrease in the cell growth of green-light illuminated *Synechocystis*/pKTLYS culture was possibly due to cell death caused by increased cell permeability resulting from the green-light induced lysis gene products.

**Figure 3:**
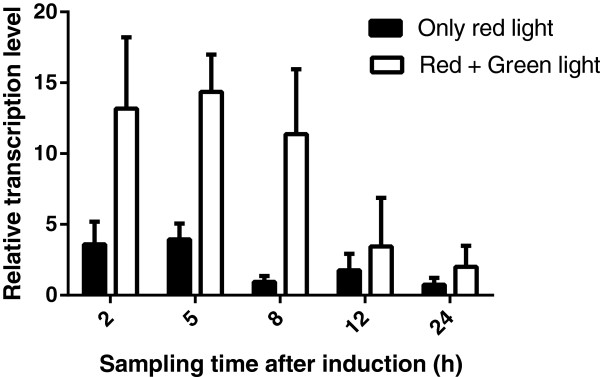
**Analysis of T4 holin transcription level by RT-PCR.** Total RNA was purified from *Synechocystis*/pKTLYS at 2, 5, 8, 12, and 24 hours after green-light induction to analyze the relative transcription level of the holin gene under red light (black bars) and green light (white bars).

To confirm that the green-light sensing system induced the lysis genes, we investigated the mRNA level of T4 holin, whose transcription was designed to be regulated by the green-light activated response regulator CcaR. The culture of *Synechocystis*/pKTLYS was sampled at 2, 5, 8, 12, and 24 hours after starting green-light illumination, and the level of T4 holin mRNA was analyzed by reverse transcription PCR (RT-PCR) (Figure 3). The transcriptional level of T4 holin after initiating green-light illumination was approximately threefold higher than those of cells cultivated under only red-light illumination. After 24 hours, the transcription level under green light decreased down to the basal mRNA level observed under red light. The T4 holin gene was therefore regulated under the green-light sensing system, and was induced by green-light illumination prior to the observable phenotypic changes, such as the leakage of phycocyanin and decrease of cell growth.

These results indicated that our constructed green-light induced lytic system was repressed under the cultivation conditions with red-light illumination, and was clearly induced under green-light illumination, resulting in lytic cells that released intracellular phycocyanin into the culture supernatant.

### Characterization of lytic cyanobacterial cells

**Figure 4:**
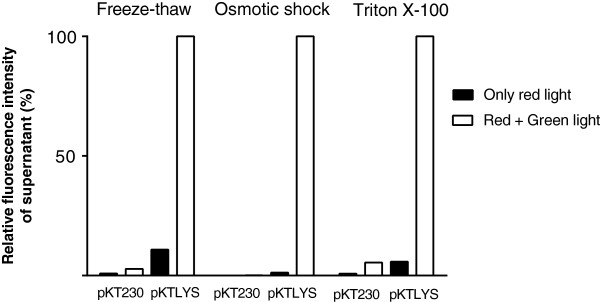
**Characterization of lytic cyanobacterial cells under different stress treatments.***Synechocystis*/pKT230 (pKT230) and *Synechocystis*/pKTLYS (pKTLYS) were incubated for 64 hours under only red light (black bars) or under simultaneous red and green light (white bars). Cells were then subjected to freeze-thaw, osmotic shock, or Triton X-100 treatments, followed by measurement of the supernatant fluorescence intensity, representing the released phycocyanin.

**Figure 5:**
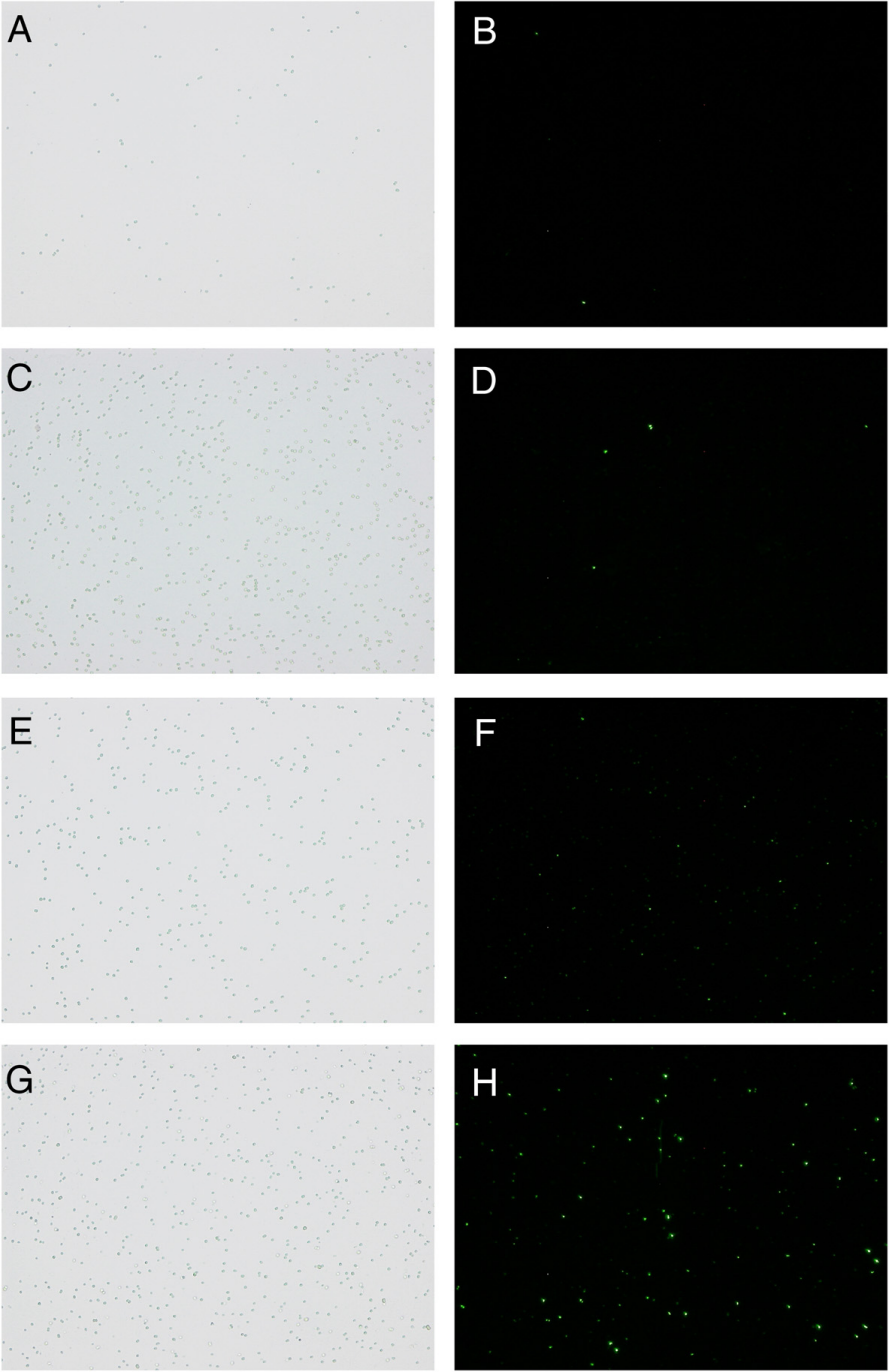
**Microscopic images of SYTOX green-stained*Synechocystis*.***Synechocystis*/pKT230 grown under continuous red light **(A and B)** or under red and green light **(C and D)**, and *Synechocystis*/pKTLYS grown under red light **(E and F)** or under red and green light **(G and H)** were observed by visible-light microscopy **(A, C, E, and G)** and by fluorescence microscopy **(B, D, F, and H)**. All cells were grown for 64 hours and then incubated with 5 μM SYTOX Green nucleic acid stain for five minutes at room temperature. Fluorescence microscopy was performed on a Biozero BZ*-*8000 (Keyence, Osaka, Japan) (excitation: 485 nm, emission: 520 nm).

The prepared lytic cells of *Synechocystis*/pKTLYS expressing lysis genes under green-light illumination were then characterized by monitoring leakage of phycocyanin under various treatments to confirm their increased cell fragility, such as detergent (Triton X-100), freeze-thaw, or osmotic shock treatments (Figure 4). The condition of the *Synechocystis* plasma membrane was also investigated by SYTOX Green nucleic acid staining (Life technologies, MD, United States) (Figure 5).

Triton X-100 treatment did not cause significant leakage of phycocyanin from *Synechocystis*/pKT230 cells to the supernatant. However, Triton X-100 treatment caused cells from *Synechocystis*/pKTLYS induced under green light to release over 20-fold more phycocyanin than *Synechocystis*/pKTLYS cultivated under continuous red light. Similarly, osmotic-shock and freeze-thaw treatments resulted in 1.2 and 11%, respectively, of phycocyanin release to the supernatant from *Synechocystis*/pKTLYS cultivated under continuous red-light illumination compared to red and green light.

SYTOX Green is a fluorescent indicator dye commonly used to assess cell death by tightly binding to nucleic acids in cells whose plasma membranes have lost integrity [26]. Microscopic analysis revealed that only 2.7 ± 1.7% and 3.3 ± 3.3% of the *Synechocystis*/pKT230 cells cultivated under continuous red-light illumination and under green-light illumination, respectively, were stained with SYTOX Green. Comparable results were observed with the non-induced *Synechocystis*/pKTLYS, where only 5.7 ± 2.3% of the cells were stained. In sharp contrast, 39 ± 19% of the *Synechocystis*/pKTLYS cells cultivated under green light were stained, indicating that a considerable fraction of the cells had compromised membranes as a result of the green-light induced lysis genes.

These results demonstrate that the green-light induced lytic system caused partial-cell lysis, rendering the cell membranes fragile. These cells could then easily be further disrupted under mild stress, such as osmotic shock or incubation with detergent. Thus, prepared lytic cyanobacterial cells should be beneficial for the recovery of biofuels and related compounds from cells, since the fragile nature of the induced cells helps to minimize the energy needed to disrupt the cell wall. Moreover, the fact that the developed system is induced by a signal transduction system responding to a physical signal (a change in the wavelength of light) is definitely advantageous over chemical induction, which results in the culture medium remaining contaminated by the chemical inducer. One of the important issues to be addressed before realizing the microalgal bioprocesses for biofuels is the minimization of water used for cultivation; the recycling of the culture medium is therefore ideal. However, compounds used in chemical-based signal transduction systems are technically quite difficult to remove or recover, and are also energetically unfavorable for industrial applications. In practice, cyanobacterial cells will be cultured under continuous illumination of sunlight with a filter to cut out green light, and will then be easily induced by removing the filter, allowing all wavelengths of light, including green light, to reach the cyanobacterial cells.

Another potential beneficial feature of the green-light inducible lytic system is that cell growth slows down greatly. The SYTOX Green staining experiment indicated that the system can be used to induce cell death by green-light exposure. This property suggests a future possibility to construct a photosynthetic genetically modified organism (GMO) that is unable to survive under sunlight exposure. Although the system still needs to be improved, we have demonstrated a potential future platform concept for photoautotrophic GMOs unable to survive if accidentally released into the natural habitat.

## Conclusions

We succeeded in genetically engineering a green-light inducible lytic system in *Synechocystis*. Illumination of the engineered cyanobacteria resulted in a considerably reduced growth rate and we demonstrated using osmotic shock, freeze-thaw, and detergent treatments, as well as by SYTOX Green staining, that the plasma membranes were compromised. The use of light-sensing two-component systems to regulate the expression of exogenous genes in cyanobacteria promises to replace conventional chemical inducers in many bioprocess applications, impacting the limiting water management issues. The ability to use such a system to regulate cyanobacterial lysis also has the potential to replace the inconvenient and expensive mechanical cell disruption methods. The proposed system demonstrated a potential future platform concept for photoautotrophic GMOs that are unable to survive under sunlight if accidentally released in the natural habitat.

## Methods

### Plasmids and strains

All gene constructs were created by standard DNA manipulations using *Escherichia coli* DH5α. Using overlap extension PCR, we constructed the green-light inducible lysis cassette containing the following in order: (1) P_cpcG2_, the region containing the promoter regulated by CcaS-CcaR green-light sensor; (2) T4 holin gene; (3) T4 endolysin gene; (4) double transcriptional terminator (BioBrick part BBa_B0015) (BioBrick foundation, MA, United State); (5) constitutive promoter (BioBrick part BBa_J23116); (6) antiholin gene; and (7) double transcriptional terminator (BioBrick part BBa_B0015). To increase the expression level of the T4 holin gene, the exogenous Shine-Dalgarno-like sequence from the upstream region of the *cpcB* gene of *Synechococcus* sp. PCC 7002 (5′-TAGGAGATAAAAATA-3′) was inserted upstream of the start codon of the T4 holin gene by inverse PCR [27]. The lysis cassette (entire sequence and gene organization provided in Additional files 1 and 2) was inserted into the *Pst*I sites of the RSF1010-derived broad host range vector pKT230, removing the kanamycin resistance gene and keeping the streptomycin resistance gene. All primers used for constructing the lysis cassette are listed in Additional file 3. *Synechocystis* was grown with gentle shaking (100 rpm) in 100 mL Erlenmeyer flasks containing 40 mL BG11 medium (0% NaCl) [28] supplemented with 10 mM TES–KOH (pH 7.4). *Synechocystis* transformed with pKT230-derived vectors were grown in the same medium supplemented with 50 μg/mL streptomycin. Cyanobacterial cells were cultured at 30°C under continuous overhead illumination provided by red-light (620 nm) light-emitting diode (Panasonic, Osaka, Japan) at an intensity of 20 μmol m^-2^ s^-1^. For gene induction, the cultures were also illuminated from below by green-light (520 nm) light-emitting diode (NK System, Osaka, Japan) at an intensity of 20 μmol m^-2^ s^-1^.

### Transformation of *Synechocystis*

A 40 mL linear phase culture (Optical density at 730 nm (OD_730_) = 0.4) of *Synechocystis* was harvested by centrifugation. The pellet was washed three times with decreasing volumes (30 mL, 20 mL, and 15 mL) of 1 mM HEPES buffer and re-suspended in 200 μL of the remaining liquid. For electroporation, 40 μL of the suspended cells were mixed with 500 ng plasmid DNA inside a 2 mm cuvette and pulsed with a Gene Pulser Xcell (Biorad, CA, United States) at 12 kV/cm at a time constant of 5 ms. Immediately following the pulse, 1 mL of BG11 was added into the cuvette and transferred to 5 mL BG11 in an Erlenmeyer flask to undergo a one-day recovery at 30°C under red-light illumination (660 nm, 20 μmol m^-2^ s^-1^). The cells were then concentrated by centrifugation to 500 μL, mixed with 5 mL melted BG11 soft agar, and spread onto a BG11 plate containing 50 μg/mL streptomycin). The plate was incubated at 30°C under red light and transformed colonies were confirmed by PCR.

### Lysis evaluation in *Synechocystis*

*Synechocystis* transformed with the pKT230 empty vector or pKT230 harboring the lysis cassette, respectively *Synechocystis*/pKT230 and *Synechocystis*/pKTLYS, were inoculated into 40 mL BG11 liquid medium at OD_730_ = 0.1 and grown under red light as described above. When the transformants reached exponential growth, at OD_730_ near 0.5, half the cultures were kept under red light and the other half were transferred to simultaneous red- and green-light illumination, as described above. The samples were then monitored for two days by measuring the OD_730_ of the cultures and the fluorescence intensity (excitation: 620 nm, emission: 650 nm) of the supernatant after centrifugation (4,000 × *g*, 2 min.) using a plate reader (Thermo Fisher Scientific Inc., MA, United States). All conditions were investigated in triplicate.

### RNA extraction and transcription analysis of holin by RT-PCR

Total RNA was isolated from 25 mL of culture using NucleoSpin RNA Clean-up kit (Takara Bio Inc., Shiga, Japan) according to the manufacturer’s instructions and treated with DNase to remove genomic DNA. The extracted RNA was reverse transcribed to cDNA using PrimeScript® RT reagent kit with gDNA Eraser (Takara Bio Inc., Shiga, Japan). The transcription levels of 16S rRNA and T4 holin were analyzed by preparing reaction solutions with SYBR Premix Ex Taq™ II (Tli RNaseH Plus) (Takara Bio Inc., Shiga, Japan) using primers shown in Additional file 3. The transcription level of T4 holin was normalized by the transcription level of 16S rRNA based on the ∆∆Ct method.

### Cell fragility assay

To evaluate whether the cells expressing lysis genes were rendered fragile, the effects of osmotic shock, freeze-thaw, and detergent on cell integrity were investigated. After 64 hours of green-light induction, 1 mL of green-light illuminated or green- and red-light illuminated *Synechocystis*/pKT230 and *Synechocystis*/pKTLYS were harvested by centrifugation. The osmotic-shock treatment was carried out by re-suspending the harvested pellet in 1 mL deionized water and incubating 1 hour at room temperature. The effect of freeze-thaw was investigated by placing the harvested pellet in –80°C for 30 minutes followed by 30°C for 20 minutes. This freeze-thaw was repeated once and the pellet was then re-suspended in fresh BG11 medium. The detergent treatment was carried out by re-suspending the harvested pellet in BG11 containing 1% Triton X-100 and incubating for 1 hour at room temperature. All treated samples were then centrifuged (4,000 × g, 2 min) and the fluorescence intensity (excitation: 620 nm, emission: 650 nm) of the supernatant measured.

## Abbreviations

GMO: Genetically modified organism; OD: Optical density; PCR: polymerase chain reaction; RT-PCR: Reverse transcription PCR; Synechocystis: *Synechocystis* sp. PCC 6803.

## Competing interests

The authors declare that they have no competing interests.

## Authors’ contributions

KM performed the evaluation of lysis systems, KA constructed the vectors used in this work, MNakamura evaluated the effect of introducing SD-like sequence in front of ATG for green-light induction. MNakajima, SF, KK, KI, and KS performed data analysis. KM, WY, KA, SF, KI, and KS participated in its design and coordination and wrote the manuscript. All authors drafted and revised the work. All authors read and approved the final manuscript.

## Supplementary Material

Additional file 1 **The cyanobacterial lysis device pKTLYS (bottom) was created by inserting lysis genes into the broad-host-range vector pKT230 (top).** The promoter region of the *Synechocystis* cpcG2 gene (recognized by the CcaR response regulator) was inserted upstream of the T4 holin and T4 endolysin genes. T4 antiholin is constitutively expressed under a weak constitutive promoter (BioBrick BBa_J23116). The cassette containing the above lysis genes was inserted into the PstI sites of pKT230 to create pKTLYS. Click here for file

Additional file 2 **Sequence of T4 lysis cassette that was inserted into the PstI sites of pKT230 to create pKTLYS, used in this study.** The cpcG2 promoter region (green) and an SD-like sequence (red) of the *Synechococcus* sp. PCC 7002 cpcB gene were inserted upstream of the T4 holin (blue) and T4 endolysin (orange) genes. T4 antiholin (pink) is constitutively expressed. Click here for file

Additional file 3 **List of primers used in this work.**[13]. Click here for file
