# Supplementary figures and images for: A green-light inducible lytic system for cyanobacterial cells
Source: Biotechnol Biofuels. 2014 Apr 9;7:56. doi: 10.1186/1754-6834-7-56 (PMC4021604; doi:10.1186/1754-6834-7-56)

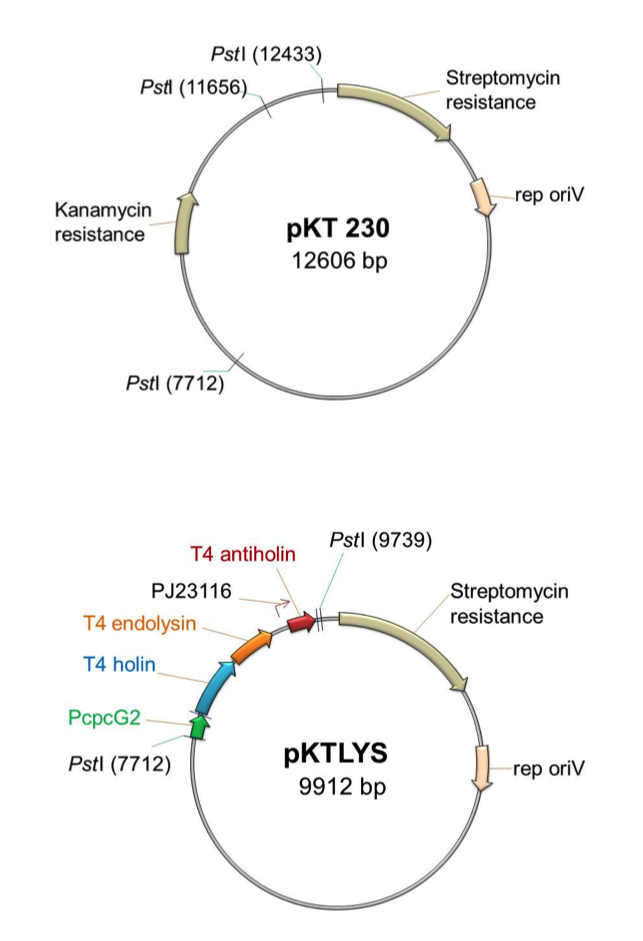

Supplement: Additional file 1 — The cyanobacterial lysis device pKTLYS (bottom) was created by inserting lysis genes into the broad-host-range vector pKT230 (top). The promoter region of the Synechocystis cpcG2 gene (recognized by the CcaR response regulator) was inserted upstream of the T4 holin and T4 endolysin genes. T4 antiholin is constitutively expressed under a weak constitutive promoter (BioBrick BBa_J23116). The cassette containing the above lysis genes was inserted into the PstI sites of pKT230 to create pKTLYS. [file 1754-6834-7-56-S1.png]
