# Supplementary material for: A green-light inducible lytic system for cyanobacterial cells
Source: Biotechnol Biofuels. 2014 Apr 9;7:56. doi: 10.1186/1754-6834-7-56 (PMC4021604; doi:10.1186/1754-6834-7-56)
Supplement: Additional file 2 — Sequence of T4 lysis cassette that was inserted into the PstI sites of pKT230 to create pKTLYS, used in this study. The cpcG2 promoter region (green) and an SD-like sequence (red) of the Synechococcus sp. PCC 7002 cpcB gene were inserted upstream of the T4 holin (blue) and T4 endolysin (orange) genes. T4 antiholin (pink) is constitutively expressed. [file 1754-6834-7-56-S2.docx]

**Additional file 2**

**Sequence of T4 lysis cassette that was inserted into the PstI sites of pKT230 to create pKTLYS, used in this study. The cpcG2 promoter region (green) and an SD-like sequence (red) of the Synechococcus sp. PCC 7002 cpcB gene were inserted upstream of the T4 holin (blue) and T4 endolysin (orange) genes. T4 antiholin (pink) is constitutively expressed.**

**TCCTCCACTAAAAGAATTCTCATAGCCCATTGTGCTTTTCTCTATCAACC**

**TCAGCTTACCTGAAGGGGTGAACAGGTCTGGGTTAATTCATGTTGCGAAA**

**TGTAACAGTTTTAGTCGCATCAGCTAACTTTCCGATTTCTTTACGATTTT**

**CTCCCCCTTTTCTTCAATTTTACTTTGTTAGGATCGCATTTTTAATGCCA**

**ACACATACCAGTTATTGGCTGGACATTAAACAACTTTTAAGTTTAATTAC**

**TAACTTTATCtataagtaggagataaaaa**T**atggcagcacctagaatatc**

**attttcgccctctgatattctatttggtgttctcgatcgcttgttcaaag**

**ataacgctaccgggaaggttcttgcttcccgggtagctgtcgtaattctt**

**ttgtttataatggcgattgtttggtataggggagatagtttctttgagta**

**ctataagcaatcaaagtatgaaacatacagtgaaattattgaaaaggaaa**

**gaactgcacgctttgaatctgtcgccctggaacaactccagatagttcat**

**atatcatctgaggcagactttagtgcggtgtattctttccgccctaaaaa**

**cttaaactattttgttgatattatagcatacgaaggaaaattaccttcaa**

**caataagtgaaaaatcacttggaggatatcctgttgataaaactatggat**

**gaatatacagttcatttaaatggacgtcattattattccaactcaaaatt**

**tgcttttttaccaactaaaaagcctactcccgaaataaactacatgtaca**

**gttgtccatattttaatttggataatatctatgctggaacgataaccatg**

**tactggtatagaaatgatcatataagtaatgaccgccttgaatcaatatg**

**tgctcaggcggccagaatattaggaagggctaaataa**ttatactagagat

acttaggaggtatt**atgaatatatttgaaatgttacgtatagatgaaggt**

**cttagacttaaaatctataaagacacagaaggctattacactattggcat**

**cggtcatttgcttacaaaaagtccatcacttaatgctgctaaatctgaat**

**tagataaagctattgggcgtaattgcaatggtgtaattacaaaagatgag**

**gctgaaaaactctttaatcaggatgttgatgctgctgttcgcggaatcct**

**gagaaatgctaaattaaaaccggtttatgattctcttgatgcggttcgtc**

**gctgtgcattgattaatatggttttccaaatgggagaaaccggtgtggca**

**ggatttactaactctttacgtatgcttcaacaaaaacgctgggatgaagc**

**agcagttaacttagctaaaagtagatggtataatcaaacacctaatcgcg**

**caaaacgagtcattacaacgtttagaactggcacttgggacgcgtataaa**

**aatctataa**agctactagagccaggcatcaaataaaacgaaaggctcagt

cgaaagactgggcctttcgttttatctgttgtttgtcggtgaacgctctc

tactagagtcacactggctcaccttcgggtgggcctttctgcgtttatat

actagagttgacagctagctcagtcctagggactatgctagctactagag

gataggaggccttt**atggccttaaaagcaacagcactttttgccatgcta**

**ggattgtcatttgttttatctccatcgattgaagcgaatgtcgatcctca**

**ttttgataaatttatggaatctggtattaggcacgtttatatgctttttg**

**aaaataaaagcgtagaatcgtctgaacaattctatagttttatgagaacg**

**acctataaaaatgacccgtgctcttctgattttgaatgtatagagcgagg**

**cgcggagatggcacaatcatacgctagaattatgaacattaaattggaga**

**ctgaatga**aattactagagccaggcatcaaataaaacgaaaggctcagtc

gaaagactgggcctttcgttttatctgttgtttgtcggtgaacgctctcT

ACTAGTAGCGGCCGCTGCAGtccggcaaaaaaacgggcaaggtgtcacca

ccctgccctttttctttaaaaccgaaaagattacttcgcgttatgcaggc

ttcctcgctcactgactcgctgcgctcggtcgttcggctgcggcgagcgg

tatcagctcactcaaaggcggtaat
