# Supplementary material for: A green-light inducible lytic system for cyanobacterial cells
Source: Biotechnol Biofuels. 2014 Apr 9;7:56. doi: 10.1186/1754-6834-7-56 (PMC4021604; doi:10.1186/1754-6834-7-56)
Supplement: Additional file 3 — List of primers used in this work.[13]. [file 1754-6834-7-56-S3.docx]

**Additional file 3 List of primers used in this work**

| Name of primer | Sequence (5′ to 3′) |
| --- | --- |
| RT-PCR 16S FW | GCTAATACCCAATGTGCCGA |
| RT-PCR 16S Rev | CACTGCTGCCTCCCGTAG |
| RT-PCR holin Fw | ATGGCAGCACCTAGAATATCA |
| RT-PCR holin Rv | CTTATAGTACTCAAAGAAACTATCTCCCC |
| Clone PcpcG2 Fw [Ref 1] | CCCCTTTTCTTCAATTTTACTT |
| Clone PcpcG2 Rv [Ref 1] | TTCGTTGATGAAGCCAGGT |
| Overlap-Fw1 | AACTGCAGTCCTCCACTAAAAGAATTCTCATAGCCC |
| Overlap-Rv1 | GTGCTGCCATGATAAAGTTAGTAATTAAACTTAAAAGTTGT |
| OverlapPCR-Fw2 | TAACTTTATCATGGCAGCACCTAGAATAT |
| OverlapPCR-Rv2 | CTGCAGGGACAACAGATAAAACGAAAGGCCCA |
| T4holin-SD Fw | CTACTTATAAGATAAAAATATGGCAGCACCTAGAATATCAT |
| T4holin-SD Rv | CCTACTTATAGATAAAGTTAGTAATTAAACTTAAAAGTTGTTT |
| PstI-lysis-PstI Fw | AACTGCAGTCCTCCACTAAAAGAATTCTCATAGCCC |
| PstI-lysis-PstI Rv | CTGCAGGGACAACAGATAAAACGAAAGGCCCA |
| pKT230-seq1 | GCTCCATAGGCCGCTTTC |
| pKT230-seq2 | GTGCGGATGAAGTCAGC |

Reference 1: Hirose Y, Shimada T, Narikawa R, Katayama M, Ikeuchi M: **Cyanobacteriochrome CcaS is the green light receptor that induces the expression of phycobilisome linker protein.** *Proc. Natl. Acad. Sci. USA* 2008, **105**, 9528-9533
